# Trend analysis and epidemiological forecasting of colorectal Cancer mortality among reproductive-age women in sub-Saharan Africa

**DOI:** 10.1016/j.pmedr.2025.103167

**Published:** 2025-07-07

**Authors:** Jiefeng Zhao, Daxing Miao, Tao Yang, Jiang Chen, Xiangquan Lai

**Affiliations:** a Department of Colorectal and Anal Surgery, The First Affiliated Hospital of Guizhou University of Traditional Chinese Medicine, Guiyang, Guizhou Province, China; b State Key Laboratory of Holistic Integrative Management of Gastrointestinal Cancers, Xijing Hospital of Digestive Diseases, Fourth Military Medical University, Xi'an, Shanxi Province, China

**Keywords:** Colorectal cancer, Global burden disease, Women of childbearing age, Sociodemographic Index, Sub-Saharan Africa

## Abstract

**Objective:**

In Sub-Saharan Africa (SSA), the region with the highest fertility rates globally, colorectal cancer (CRC) is emerging as a major public health challenge for women of childbearing age. This study aimed to analyze CRC mortality trends, identify key driving factors, and predict future mortality patterns among women of childbearing age in SSA.

**Methods:**

We assessed CRC mortality among women of childbearing age across four SSA subregions and 46 countries, calculating age-standardized mortality rates (ASMR) and estimated annual percentage change (EAPC). The Das Gupta decomposition method quantified the contributions of aging, population growth, and epidemiological changes. A Bayesian Age-Period-Cohort model projected mortality trends from 2022 to 2040, and an Autoregressive Integrated Moving Average model were used for sensitivity analysis.

**Results:**

From 1990 to 2021, CRC mortality rose significantly in Eastern and Western SSA. In 2021, Southern SSA had the highest ASMR and fastest growth (EAPC = 1.63), while Western SSA had the lowest ASMR. The mortality in Nigeria and Zimbabwe are of particular concern. The region exhibited an overall upward trend in CRC mortality alongside increasing Socio-demographic index. Population growth was the primary driver of increasing mortality across the four subregions and 45 countries. Projections indicated that ASMR will rise in Central and Western SSA but decline in Eastern and Southern SSA by 2040.

**Conclusions:**

Population growth is a crucial factor driving the CRC mortality of women of childbearing age in SSA. Regional disparities reflected varying epidemiological transitions and healthcare capacities. Screening programs and cancer registries are key to reducing future burdens.

## Highlights

• Assessing colorectal cancer burden among reproductive-age women in Sub-Saharan Africa.
• High fertility rates are associated with increased colorectal cancer mortality.
• Highest mortality in Ethiopia, Nigeria; Zimbabwe had steepest rise.
• The impact of population growth on colorectal cancer needs to be taken seriously.

## 1 Introduction

Colorectal cancer (CRC) is a significant global health burden, ranked as the third most commonly diagnosed cancer and the second leading cause of cancer-related deaths worldwide (Sung et al., 2021). While the incidence and mortality of CRC have been declining in high-income countries due to advancements in screening and treatment, the opposite trend has been observed in low- and middle-income countries, particularly in Sub-Saharan Africa (SSA) (Arnold et al., 2017). This region, which has the highest total fertility rate, faces unique challenges in addressing non-communicable diseases, including CRC, amid persistent infectious diseases and limited healthcare resources (Bray et al., 2012).

Women of childbearing age (typically defined as ages 15–49) in SSA are particularly vulnerable to the dual burden of reproductive health issues and rising non-communicable diseases (Abualkhair et al., 2020). The intersection of high fertility rates and increasing CRC incidence poses a complex public health challenge, as these women often experience delayed diagnoses, limited treatment access, and higher mortality rates compared to women in high-income regions (Meng et al., 2025). Furthermore, the lack of region-specific data and predictive analytical models for CRC burden in this population hampers the development of targeted interventions and evidence-based policies (Ferlay et al., 2018).

By leveraging the Global Burden of Disease (GBD) 2021 dataset, this study aims to analyze current CRC mortality among women of childbearing age in SSA, identify key determinants influencing these trends, and develop predictive models to estimate future mortality rates under different scenarios. Understanding these dynamics is critical for informing evidence-based strategies to reduce CRC mortality and improve the overall health outcomes for women in this region (Jemal et al., 2011).

This study focuses on unique vulnerable groups in the context of high fertility rates, and the findings will provide valuable insights for policymakers, healthcare providers, and researchers aiming to address the rising burden of CRC in SSA and other similar settings.

## 2 Methods

### 2.1 Data source

The GBD 2021 provides an extensive examination of the impacts of 371 distinct health conditions across 204 countries and territories, covering a broad spectrum of health metrics such as disease incidence, prevalence, Disability-Adjusted Life Years, mortality, risk factors, and other related indicators (*Lancet*, 2024). The compilation of data for GBD 2021 was conducted through a rigorous collection process, sourcing information from diverse databases, including national censuses, household surveys, civil registration systems, vital statistics, disease registries, health service records, air quality monitoring systems, satellite imagery, and other relevant health data repositories (*Lancet*, 2020). The data used in this study are all publicly accessible through the GBD 2021 results tool at http://ghdx.healthdata.org/gbd-results-tool. The Sociodemographic Index (SDI) was utilized to enhance the analysis and quantitatively evaluate a nation or region's overall socioeconomic development, with classifications into five tiers: low, low-middle, middle, high-middle, and high SDI levels.

SSA region was further stratified into four distinct areas: Central, Eastern, Western, and Southern SSA.

We stratified the reproductive age groups into finer categories (15–19, 20–24, 25–29, 30–34, 35–39, 40–44, and 45–49 years) to capture detailed mortality trends.

### 2.2 Analytical Methods

To analyze trends in age-standardized mortality rates (ASMR) over the study period, we calculated the estimated annual percentage change (EAPC) and used linear regression models to determine its 95 % confidence intervals (CIs) (Hankey et al., 2000).

### 2.3 Statistics

The Das Gupta decomposition method was used to attribute mortality changes to three factors: aging, population growth, and epidemiology. This methodology quantifies the contribution of various factors to observed changes by algebraically isolating the standardized effects of each contributing multiplicative factor (Das Gupta, 1978).

In this study, we applied the Bayesian Age-Period-Cohort model to project the mortality rate of CRC among women of childbearing age in SSA from 2022 to 2040. For sensitivity analysis, we employed an Autoregressive Integrated Moving Average model. All data processing, statistical analysis, and visualization were performed using R (version 4.4.2) and the JD_GBDR software (V2.37, Jingding Medical Technology Co., Ltd.).

## 3 Results

### 3.1 Mortality trends of CRC among women of childbearing age in SSA subregions

Among the four SSA subregions, Eastern SSA had the highest number of CRC deaths among women of childbearing age in both 1990 (510.76 cases; 95 % Uncertainty Interval: 341.31–633.62) and 2021 (1048.02 cases; 95 % Uncertainty Interval: 855.91–1328.70). In contrast, Central SSA recorded the lowest mortality burden, with 99.59 cases (95 % Uncertainty Interval: 70.76–135.82) in 1990 and 270.97 cases (95 % Uncertainty Interval: 183.09–382.10) in 2021. Eastern SSA showed the largest absolute increase in deaths (+537.32 cases), while Western SSA experienced the greatest percentage change (187.52 %) over the study period (Table 1).

**Table 1:** Deaths and age-standardized mortality rate of colorectal cancer among women of childbearing age in 1990 and 2021 and estimated annual percentage change from 1990 to 2021 in Sub-Saharan Africa Subregions.

Location	Death cases-1990 No. (95 %UI)	1990-ASMR-per 100,000 (95 % UI)	Death cases-2021 No. (95 %UI)	2021-ASMR-per 100,000 (95 % UI)	EAPC (95 % CI)
Sub-Saharan Africa	1039.61(814.72,1230.04)	0.92(0.72,1.09)	2408.70(1994.13,2959.41)	0.86(0.71,1.05)	−0.22(−0.30,-0.13)
Central Sub-Saharan Africa	99.59(70.76,135.82)	0.81(0.57,1.10)	270.97(183.09,382.10)	0.83(0.56,1.17)	0.15(0.05,0.24)
Eastern Sub-Saharan Africa	510.76(341.31,633.62)	1.18(0.79,1.47)	1048.02(855.91,1328.70)	0.98(0.80,1.24)	−0.94(−1.11,-0.76)
Western Sub-Saharan Africa	242.38(192.19,302.18)	0.56(0.44,0.69)	696.67(499.26,934.73)	0.58(0.42,0.78)	0.33(0.27,0.40)
Southern Sub-Saharan Africa	186.87(165.63,213.21)	1.41(1.25,1.60)	393.04(327.85,471.79)	1.81(1.51,2.17)	1.63(1.06,2.19)

Abbreviations: ASMR, age-standardized mortality rate; EAPC, estimated annual percentage change; UI, Uncertainty Interval; CI, Confidence Interval.

**Fig. 1:**
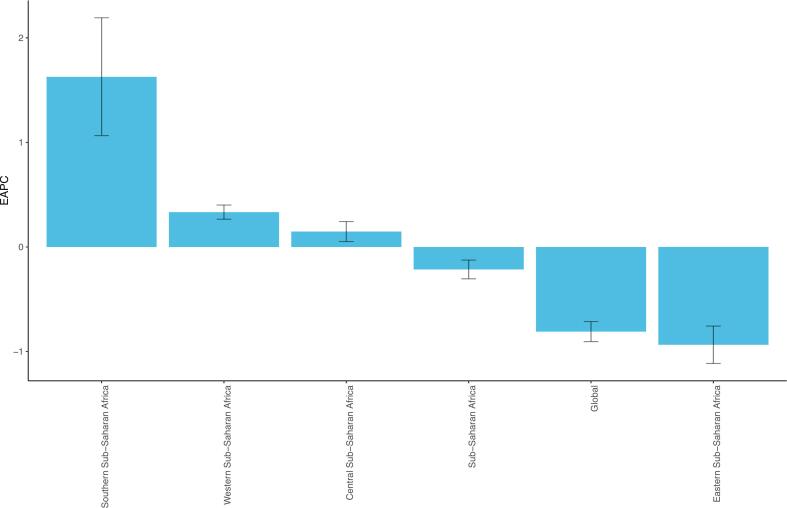
The estimated annual percentage change of age-standardized mortality rate of colorectal cancer among women of childbearing age in global, Sub-Saharan Africa and four subregions from 1990 to 2021. Abbreviations: EAPC: estimated annual percentage change. Note: The age-standardized mortality rate adjusts the mortality rates of different age groups, eliminating the influence of varying age structures on mortality comparisons. The EAPC represents the average annual percentage change in the age-standardized mortality rate over the period from 1990 to 2021.

In 1990 and 2021, Southern SSA had the highest ASMR of CRC among women of childbearing age, with 1.41 per 100,000 people (95 % Uncertainty Interval: 1.25 to 1.60) and 1.81 per 100,000 people (95 % Uncertainty Interval: 1.51 to 2.17) respectively. Western SSA consistently showed the lowest ASMR, with 0.56 per 100,000 people (95 % Uncertainty Interval: 0.44 to 0.69) and 0.58 per 100,000 people (95 % Uncertainty Interval: 0.42 to 0.78) respectively. Southern SSA experienced the greatest ASMR increase (EAPC = 1.63; 95 % CI: 1.06–2.19), while Eastern SSA showed the most significant decrease (EAPC = −0.94; 95 % CI: −1.11 to −0.76) (Table 1; Fig. 1).

### 3.2 Mortality trends of CRC among women of childbearing age in the countries of SSA

Among the 46 analyzed countries, Ethiopia recorded the highest number of CRC deaths in 1990 (204.31 cases; 95 % Uncertainty Interval: 76.89–284.80), while Nigeria showed the highest deaths in 2021 (282.66 cases; 95 % Uncertainty Interval: 168.22–438.47). In contrast, Sao Tome and Principe had the lowest mortality burden in both years (1990: 0.12 cases; 2021: 0.34 cases). Nigeria exhibited the largest absolute increase in CRC deaths, whereas Zimbabwe experienced the greatest relative change (48 % increase) (Table S1).

**Fig. 2:**
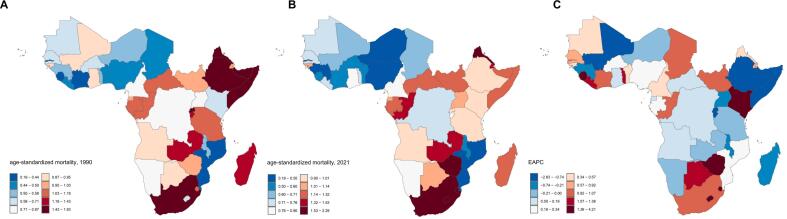
The changes of age-standardized mortality rate of colorectal cancer among women of childbearing age from 1990 to 2021 in Sub-Saharan Africa. Age-standardized mortality rate in 1990. (A) Age-standardized mortality rate in 2021. (B) Estimated annual percentage change from 1990 to 2021. Abbreviations: EAPC: Estimated annual percentage change. Note: The age-standardized mortality rate adjusts the mortality rates of different age groups, eliminating the influence of varying age structures on mortality comparisons. The EAPC represents the average annual percentage change in the age-standardized mortality rate over the period from 1990 to 2021.

Ethiopia showed the highest ASMR in 1990 (1.81 per 100,000; 95 % Uncertainty Interval: 0.68–2.52), while Zimbabwe recorded the highest rate in 2021 (2.27 per 100,000; 95 % Uncertainty Interval: 1.44–3.36). In 1990 and 2021, Mozambique maintained the lowest ASMR, with 0.19 per 100,000 people (95 % Uncertainty Interval: 0.13,0.27) and 0.19 per 100,000 people (95 % Uncertainty Interval: 0.11,0.30) respectively. Lesotho experienced the most significant ASMR increase, with an EAPC of 4.17 (95 % CI: 3.37 to 4.97). Conversely, Rwanda achieved the largest ASMR reduction, demonstrating an EAPC of −2.60 (95 % CI: −3.05 to −2.14) (Table S1 and Fig. 2).

### 3.3 Association between ASMR and SDI across SSA countries

**Fig. 3:**
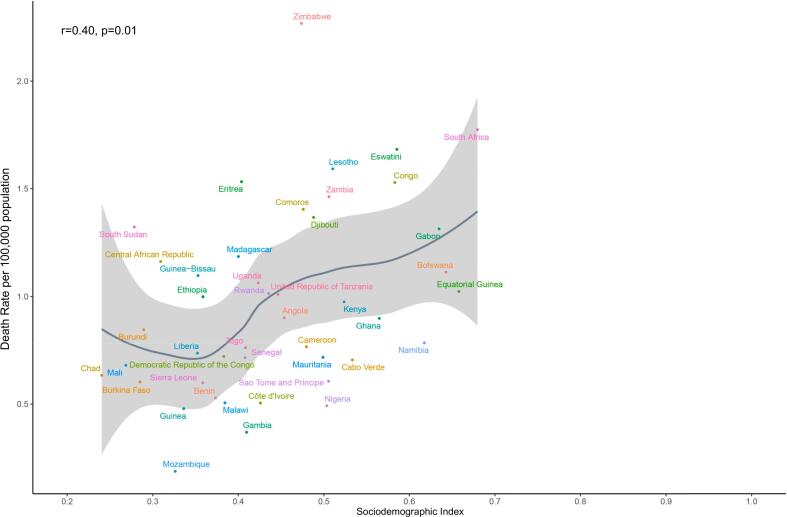
Age-standardized mortality rate of colorectal cancer among women of childbearing age for Sub-Saharan Africa countries by Sociodemographic Index, 1990–2021. Note: The Sociodemographic Index is utilized to enhance the analysis and quantitatively evaluate a nation or region's overall socioeconomic development, with classifications into five tiers: low, low-middle, middle, high-middle, and high SDI levels.

In 2021, our analysis revealed a positive correlation (*r* = 0.40, *p* = 0.01) between ASMR of CRC among women of childbearing age and the SDI across 46 countries in SSA (Fig. 3). The region demonstrated an overall upward trend in CRC mortality burden with increasing SDI levels.

### 3.4 Decomposition analysis

**Fig. 4:**
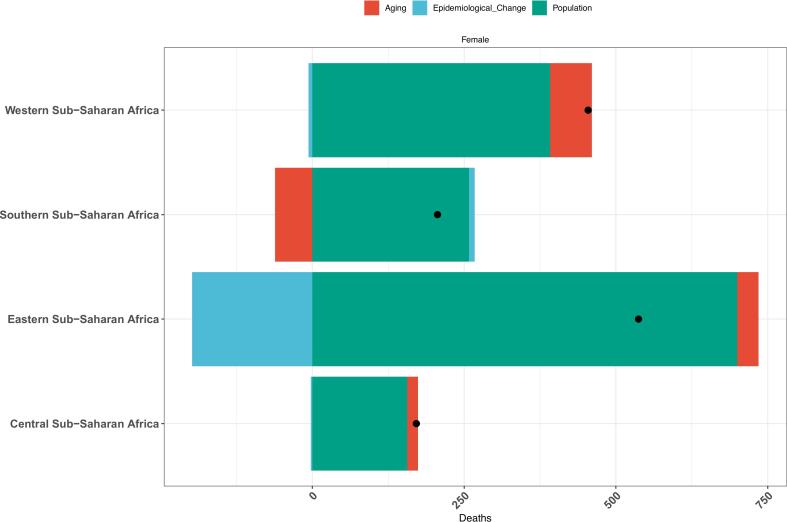
Decomposition analysis of the changes in age-standardized mortality rate of colorectal cancer among women of childbearing age across Sub-Saharan Africa subregions. Note: Aging: Contribution of population aging (shift in age structure) to age-standardized mortality rate changes. Epidemiological change: Contribution of shifts in age-specific mortality rates (e.g., healthcare interventions or disease patterns). Population: Contribution of overall population growth or decline to age-standardized mortality rate changes. Single dot interpretation: Each dot represents the observed net change in age-standardized mortality rate for a subregion, while the stacked bars show the decomposed contributions of the three factors.

We performed decomposition analysis to quantify the contributions of aging, population growth, and epidemiological changes to CRC burden among women of childbearing age across all four subregions and 46 countries. Population growth emerged as the predominant driver of increasing CRC burden in Central, Eastern, Western, and Southern SSA (Fig. 4). While population growth accounted for most of the burden increase in 45 countries, Lesotho's CRC burden was jointly driven by both epidemiological changes and population growth (Table S2; Fig. S1).

### 3.5 Prediction analysis for the burden of CRC among women of childbearing age to 2040

**Fig. 5:**
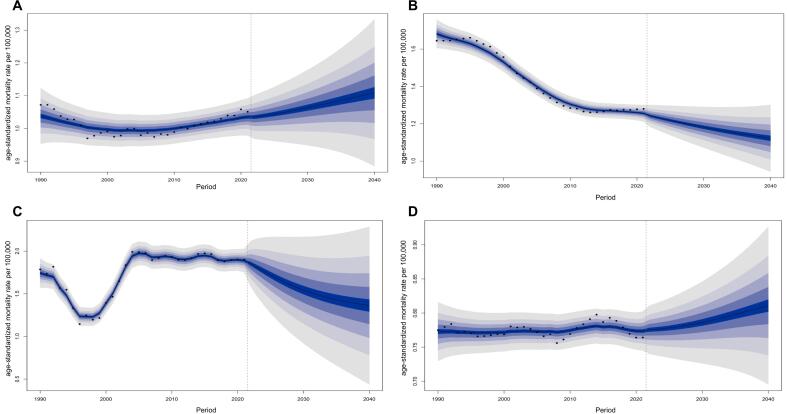
Estimated age-standardized mortality rate for colorectal cancer among women of childbearing age across Sub-Saharan Africa subregions by 2040. (A) Central Sub-Saharan Africa. (B) Eastern Sub-Saharan Africa. (C) Southern Sub-Saharan Africa. (D) Western Sub-Saharan Africa. Note: Dots represent observed historical age-standardized mortality rate data points (e.g., from 1990 to 2021). Lines show projected age-standardized mortality rate trends (2022–2040).

We applied the Bayesian Age-Period-Cohort model to analyze and predict the CRC mortality trends among women of childbearing age across four SSA regions. As shown in Fig. 5, by 2040, the ASMR of CRC among women of childbearing age was projected to increase in Central and Western SSA, while a decreasing trend was expected in Eastern and Southern SSA. For sensitivity analysis, we employed the Autoregressive Integrated Moving Average model, which predicted rising ASMR in Central and Eastern SSA from 2022 to 2040, with no significant trends in Western and Southern SSA (Fig. S2).

The study also forecasted ASMR trends for each age subgroup across these regions. From 2022 to 2040, the ASMR of CRC among women of childbearing age was projected to increase across all age subgroups in Central and Western SSA. In contrast, the ASMR was expected to decrease across all age subgroups in Eastern SSA. In Southern SSA, the ASMR was projected to increase among women aged 15–19 and 20–24 years, while it was expected to decrease among those aged 35–39, 40–44, and 45–49 years. For women aged 25–29 and 30–34 years in Southern SSA, the ASMR was predicted to initially decline and then rise (Fig. S3).

## 4 Discussion

This study systematically analyzed the spatiotemporal evolution of CRC mortality among women of childbearing age in SSA, the region with the highest fertility in the world, and projected future trends. The results uncovered significant regional disparities while providing novel insights into CRC disease burden in high-fertility, resource-limited settings.

Significant disparities in CRC mortality rates among women of childbearing age were found across subregions of SSA. For example, Eastern SSA had the highest number of deaths, with 1048 cases in 2021. In contrast, Southern SSA experienced the fastest increase in ASMR, with an EAPC of 1.63. This phenomenon may reflect two contrasting trends: On one hand, Southern SSA, including countries like South Africa and Botswana, is experiencing rapid economic development and healthcare advancements (Tinta, 2022), which have improved cancer reporting and increased patient visit rates, contributing to higher CRC mortality data (Zhang et al., 2024). Multiple studies have shown that westernized dietary patterns are associated with an increased risk of CRC. In the SSA region, with the acceleration of urbanization and the improvement of living standards, the consumption of high-fat and high-sugar diets is gradually increasing (Ramaboli et al., 2024; Vorster et al., 2014; de Bruin et al., 2021). In addition, the increase in obesity rate may also be an important factor contributing to the increase in CRC mortality rate (Tazinkeng et al., 2024; Virginie et al., 2024). The adoption of the prevalence of sedentary lifestyles has contributed to the accumulation of CRC risk factors (Graham et al., 2012). However, due to the lack of specific data for SSA, we are unable to accurately quantify the contributions of these factors. Future research needs to further explore the relationship between these lifestyle factors and CRC mortality and provide more specific evidence to support it. On the other hand, Eastern SSA, including Ethiopia and Kenya, faces limited medical resources that delay diagnosis and result in high mortality rates among women of childbearing age (Virginie et al., 2024; Aynalem et al., 2024). However, localized public health interventions, such as pilot screening programs, have led to a downward trend in ASMR (Walle et al., 2025). It is worth noting that Western SSA experienced the largest change in mortality rates, yet its ASMR remained the lowest in the region. The paradox may stem from the unique demographic structure of the region: High fertility rates have led to a rapid expansion of the childbearing female population, which has diluted the increase in ASMR. However, the absolute number of cases has surged, still placing significant pressure on the healthcare system (Morgan et al., 2023).

At the national level, Ethiopia and Nigeria have the highest number of CRC deaths among women of childbearing age, while Sao Tome and Principe has the lowest. Notably, Nigeria has the largest absolute change in the number of deaths, whereas Zimbabwe has the highest relative change in mortality rate, reaching 48 %. These findings reflect significant disparities in CRC burden among countries within the SSA region. Based on these findings, Ethiopia, Nigeria, and Zimbabwe are identified as priority areas for CRC prevention and control among women of childbearing age.

As the region with the highest fertility rate globally, a decomposition analysis of this region indicated that population growth was the core driving factor for the increase in CRC deaths among women of childbearing age. In western and southern SSA, population growth contributes up to 130.27 % and 125.05 % respectively to the increase in CRC burden, and drives the CRC burden of women of childbearing age in 45 countries except for Lesotho, far exceeding the contributions of aging and epidemiological changes. This finding challenges the traditional view that the rise in chronic disease mortality is primarily driven by risk factors (The burden of diseases, 2024). In the context of high fertility rates in SSA, the population of women of childbearing age has grown significantly between 1990 and 2021 (Bhattacharjee et al., 2024; Coulibaly et al., 2025), however, CRC screening coverage remains inadequate (Graham et al., 2012). As a result, the potential “demographic dividend” has instead been transformed into a “disease burden dividend”. This mechanism is particularly evident in Nigeria: the country experienced the highest absolute increase in CRC deaths among women of childbearing age, reaching 282 additional cases in 2021. However, its ASMR increased only slightly by 0.2 per 100,000. This suggests that the surge in case numbers is primarily driven by the expansion of the childbearing female population, rather than an increase in individual disease risk. This finding has important implications for public health strategies: in regions with rapid population growth, focusing solely on individual-level risk interventions (such as lifestyle modifications) may be insufficient. It is essential to concurrently strengthen population-based early screening programs and expand healthcare capacity (Qiu et al., 2022). It is worth noting that reproductive behavior itself may influence the risk of CRC among women of childbearing age through multiple mechanisms. First, fluctuations in hormone levels, such as elevated progesterone levels during multiple pregnancies, may modulate the gut microenvironment (Basnet et al., 2024) and potentially promote the proliferation of colonic epithelial cells (Onji et al., 2025; Nieto et al., 2025). Second, the prioritization of maternal and child health care has led to the marginalization of CRC screening for women of childbearing age in primary health care settings (Calanzani et al., 2021). Third, women of childbearing age, due to factors such as family responsibilities, may delay seeking medical help compared to men (Brand et al., 2019). Fourth, in regions with high fertility rates, women of childbearing age generally lack awareness of cancer screening, resulting in a higher proportion of late-stage diagnoses (Geremew et al., 2024). These intersecting risks suggest that CRC prevention and control for this population should be integrated into reproductive health services, such as incorporating risk screening questionnaires into prenatal checkups (Kassa et al., 2024). Based on the above findings, the implementation of family planning policies may be particularly important in reducing the risk of CRC mortality risk among women of childbearing age in SSA. Some studies have confirmed the effectiveness of certain measures in reducing female fertility rates. Beyond the use of effective modern contraceptive measures being one of the key factors in reducing fertility rates, research has also found that the fertility rate of women without formal education is significantly higher than that of educated women, and this difference persists across various regions (Olowolafe et al., 2025), highlighting the importance of promoting women's education in SSA. Furthermore, international aid efforts are also actively implementing family planning and reproductive health projects in multiple African countries, aiming to reduce local fertility rates by providing contraceptives, improving service accessibility, and promoting gender equality (Sylvia et al., 2024). However, given the influence of multiple factors such as culture, politics, and economics in SSA, reducing the female fertility rate remains a long-term challenge.

Predictions from the Bayesian Age-Period-Cohort model indicate that by 2040, the ASMR of CRC among women of childbearing age will continue to rise in Central and Western SSA. In contrast, declines may be observed in Eastern and Southern SSA. Notably, with the exception of Central SSA, the sensitivity analysis results using the Autoregressive Integrated Moving Average model differed significantly from those of the Bayesian Age-Period-Cohort model. This discrepancy arises because Autoregressive Integrated Moving Average models primarily forecast the future by extrapolating historical time trends. When data exhibit a long-term upward trend, Autoregressive Integrated Moving Average models often continue this trend into future projections. Conversely, the Bayesian Age-Period-Cohort model accounts for the interaction between age, period, and cohort effects and can identify that certain cohorts or periods exert a more significant impact on mortality. Moreover, while the Autoregressive Integrated Moving Average model might fail to capture the nonlinear effects of advances in medical technology (period effect) on mortality rates in specific age groups (age effect), the Bayesian Age-Period-Cohort model is better equipped to reflect this complex relationship. However, owing to the inherent limitations of prediction models, despite the observed heterogeneity, the Bayesian Age-Period-Cohort model currently represents a relatively robust methodology. Nevertheless, these predictions highlight three key issues: First, the Bayesian Age-Period-Cohort model relies on linear extrapolation of historical trends. Yet, the epidemiological trajectory for CRC among women of childbearing age in SSA could experience abrupt shifts driven by unique factors like HIV/Tuberculosis co-infections and alterations in gut microbiota resulting from antibiotic misuse (Shebl et al., 2012; Liu et al., 2021; Pawlowski et al., 2012). Currently, these covariates are not integrated into the model. Consequently, under present conditions, our prediction results represent the best available estimate, while the actual CRC mortality trend might diverge due to these complex influences. We propose that future research explore incorporating additional covariates (such as HIV prevalence, antibiotic consumption, etc.) to refine the predictive model. This approach would enhance its ability to accurately depict CRC mortality trends across SSA regions. Second, if other countries in SSA follow the experience of Tunisia or Mauritius and promote fecal immunochemical testing screening within the next decade, it may cause the predicted trend curve to decline earlier than anticipated (Jovanka et al., 2024). Conversely, if resources continue to be disproportionately allocated to infectious diseases, the ASMR of CRC among women of childbearing age in these countries may further increase. Third, the death registration data relied upon by the GBD 2021 have low coverage in SSA (*The lancet. HIV*, 2024), and CRC is often misdiagnosed as infectious enteritis in rural areas, which may lead to an underestimation of CRC mortality. To address these limitations, we propose the following recommendations: First, implement pilot risk-based screening programs in regions with high ASMR, such as Southern SSA, focusing on women aged 35 and above. Second, create a regional cancer registry consortium to integrate data on fertility rates, HIV prevalence, and comorbidities, thereby optimizing predictive models. Third, incorporate CRC prevention into maternal and child health services, thus leveraging existing reproductive health networks to facilitate cancer prevention education. In SSA, the healthcare system consistently faces challenges of resource constraints and prioritization of infectious diseases alongside maternal and child care. Therefore, when integrating CRC screening into reproductive health services, it is imperative to fully consider local health system capacity and resource allocation. We recommend initiating a risk-based screening pilot in high ASMR regions (e.g., Southern SSA), focusing on women aged 35 and above. Additionally, existing reproductive health networks should be leveraged to integrate CRC prevention education into pre-pregnancy checkups and maternal and child health services, thereby enhancing women's awareness of CRC risks. For high-risk age groups (e.g., 35–49 years), cost-efficient screening methods (such as fecal immunochemical testing) must be prioritized, coupled with community health education initiatives to increase early diagnosis rates (Nur et al., 2025). For younger women (aged 15–24), emerging risk factors including rising smoking rates and processed food consumption require close monitoring. However, the confidence intervals of the predictions are wide, indicating substantial uncertainty. This variability likely stems from multiple sources, such as model assumption limitations and unique epidemiological trends in SSA.

This study has several limitations: First, the data relied upon in this study may have coverage gaps in SSA, particularly in rural and resource poor areas. Poor accessibility of medical resources, limited diagnostic capabilities, incomplete health information systems, and insufficient patient seeking behavior contribute to the serious underreporting of CRC, potentially leading to a systematic underestimation of the true disease burden in the region (Graham et al., 2012; Hassen et al., 2022; Afungchwi et al., 2025). Therefore, our research findings should be interpreted with caution and, where possible, validated through further localized studies. In addition, we once again call for strengthening the construction of cancer registration systems in SSA regions to improve data quality and accuracy, and provide more reliable data support for future health policy formulation. Second, the risk of misclassification of diseases or causes of death, due to inaccurate diagnosis, limitations in inferring the cause of death, coding errors, or issues in data integration, may result in uncertain biases in disease patterns, risk factor associations, or trend analysis (Alipour and Payandeh, 2021; Laanani et al., 2023). Third, significant data sparsity in SSA, particularly for specific populations with the lowest socioeconomic status (Musa et al., 2023; Seidler et al., 2025), limits our in-depth analysis of heterogeneity within the region and the generalizability of our results. Forth, the decomposition analysis did not quantify the specific contributions of social determinants such as healthcare accessibility and screening policies, which exhibit extremely high heterogeneity in SSA (Atnafu et al., 2024; Ngepah and Mouteyica, 2024). Fifth, the study did not consider the impact of environmental factors, such as climate change, which may influence CRC mortality by altering dietary habits, including increased use of preserved or fermented foods (Tazinkeng et al., 2024; Litchman, 2025). Six, the projection for 2040 is subject to uncertainty, with wide confidence intervals indicating the range of possible outcomes. This should be considered when interpreting the results. Seventh, the inability to adjust for key contextual factors such as healthcare access, education levels, and screening infrastructure limits causal interpretation of observed associations.

## 5 Conclusion

This study analyzed the mortality trends of CRC among women of childbearing age in SSA. The findings revealed significant disparities across SSA, with population growth being a crucial factor driving the CRC mortality. Projections indicate rising mortality rates in Central and Western SSA by 2040, while declines are expected in Eastern SSA. Targeted strategies are imperative to alleviate the CRC burden among women of childbearing age.

## CRediT authorship contribution statement

**Jiefeng Zhao:** Writing – review & editing, Project administration, Funding acquisition, Data curation, Conceptualization. **Daxing Miao:** Visualization, Resources, Formal analysis. **Tao Yang:** Writing – original draft, Visualization, Methodology, Funding acquisition. **Jiang Chen:** Writing – review & editing, Funding acquisition, Formal analysis. **Xiangquan Lai:** Supervision, Project administration.

## Consent for publication

Not applicable.

## Ethics statement

This study utilized publicly available databases and did not involve human participants, and the requirement for informed consent was waived.

## Declaration of competing interest

The authors declare that they have no known competing financial interests or personal relationships that could have appeared to influence the work reported in this paper.

## Data Availability

Data will be made available on request.

## Appendix A Supplementary data

Supplementary material 1 Image 1

Supplementary material 2 Image 2

Supplementary material 3 Image 3

Supplementary material 4 Image 4

Supplementary material 5 Image 5
