# Supplementary figures and images for: Trend analysis and epidemiological forecasting of colorectal Cancer mortality among reproductive-age women in sub-Saharan Africa
Source: Prev Med Rep. 2025 Jul 7;56:103167. doi: 10.1016/j.pmedr.2025.103167 (PMC12275479; doi:10.1016/j.pmedr.2025.103167)

Aging Epidemiological\_Change Population

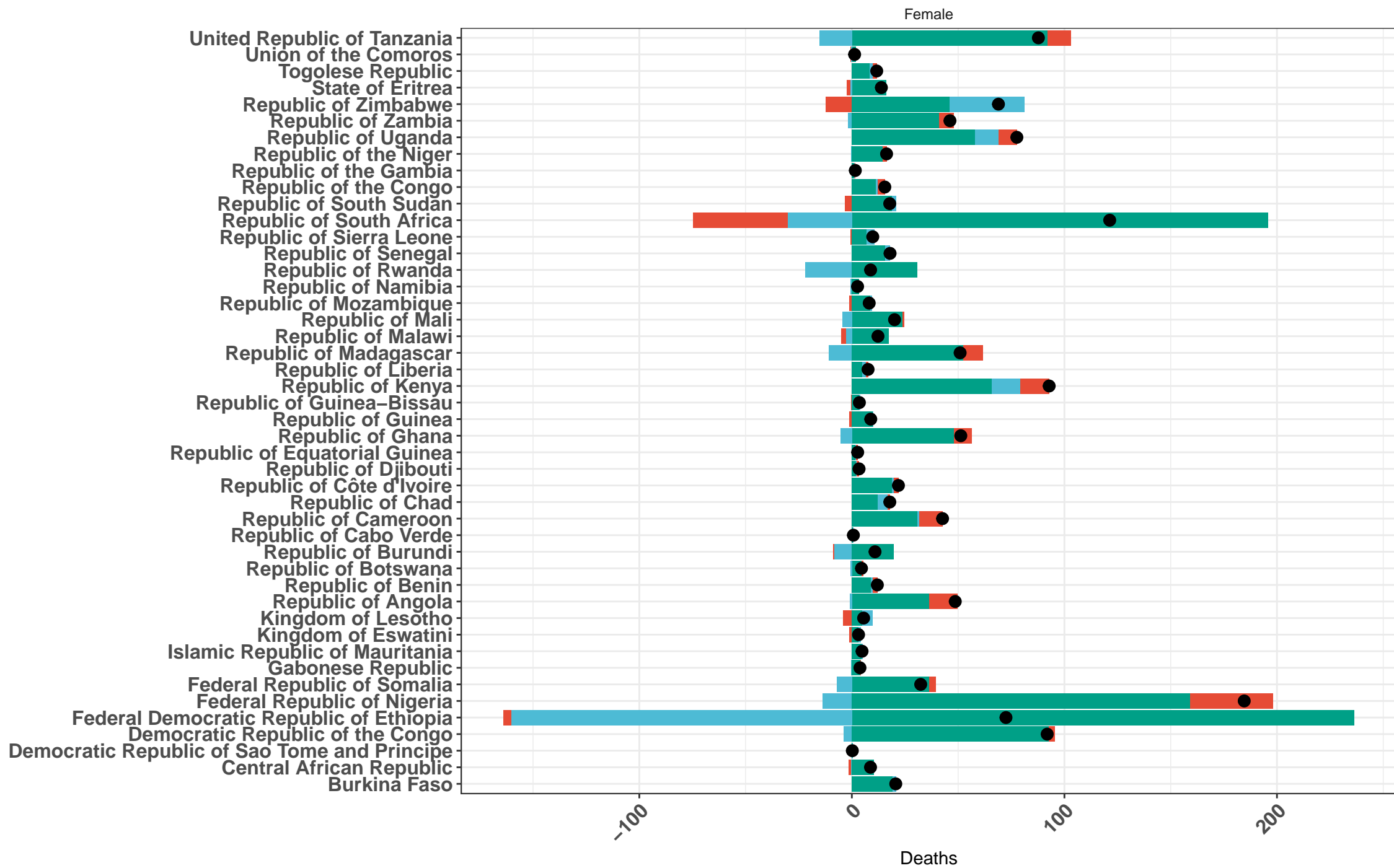

Supplement: Supplementary file 1 — Supplementary material 1 [file mmc1.pdf]

**A**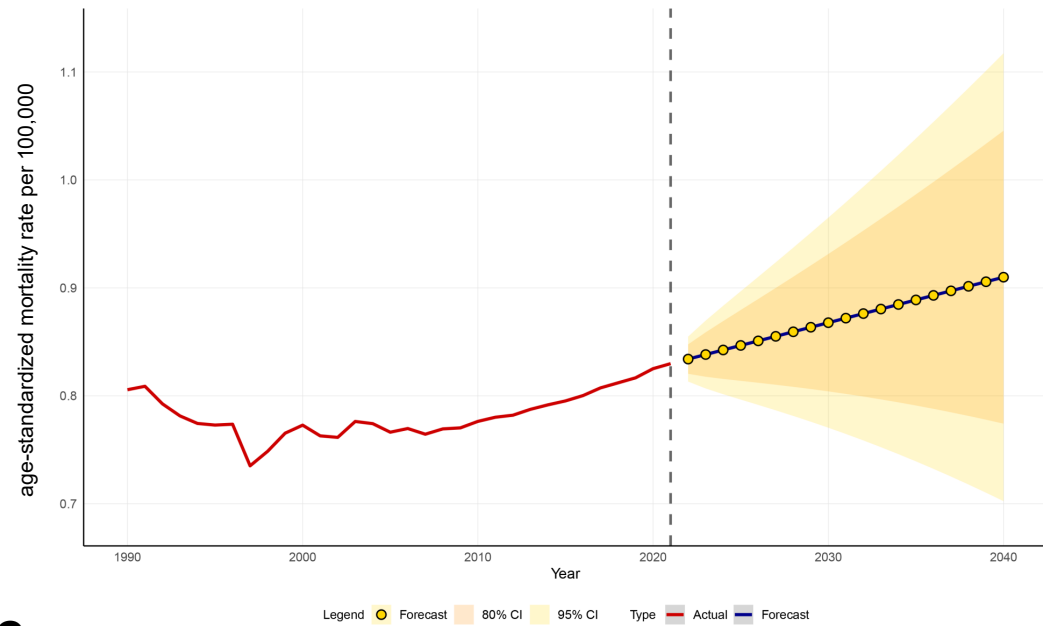**B**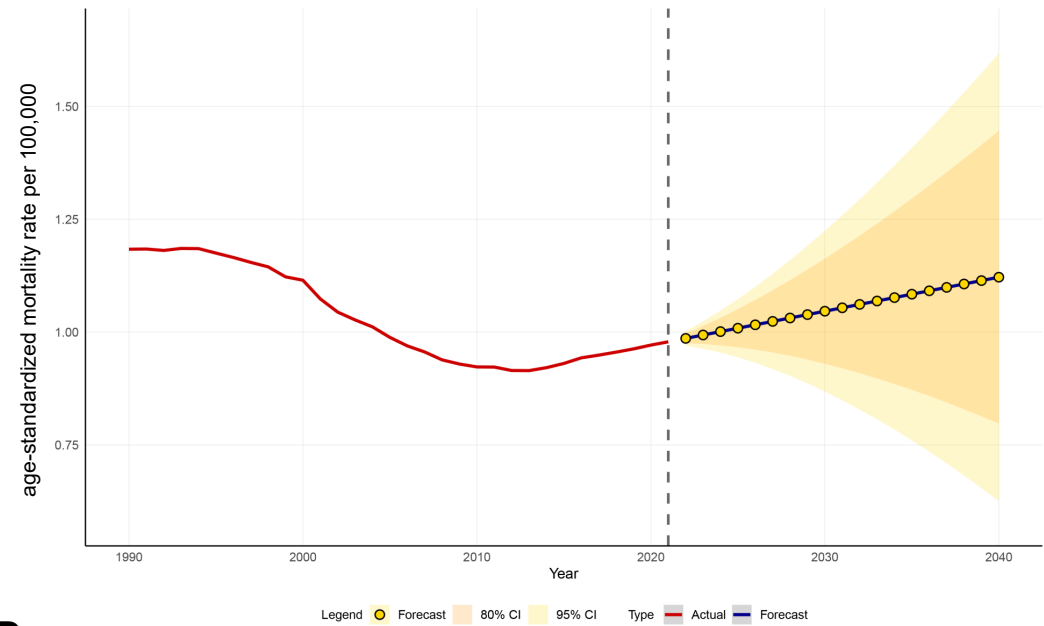**C**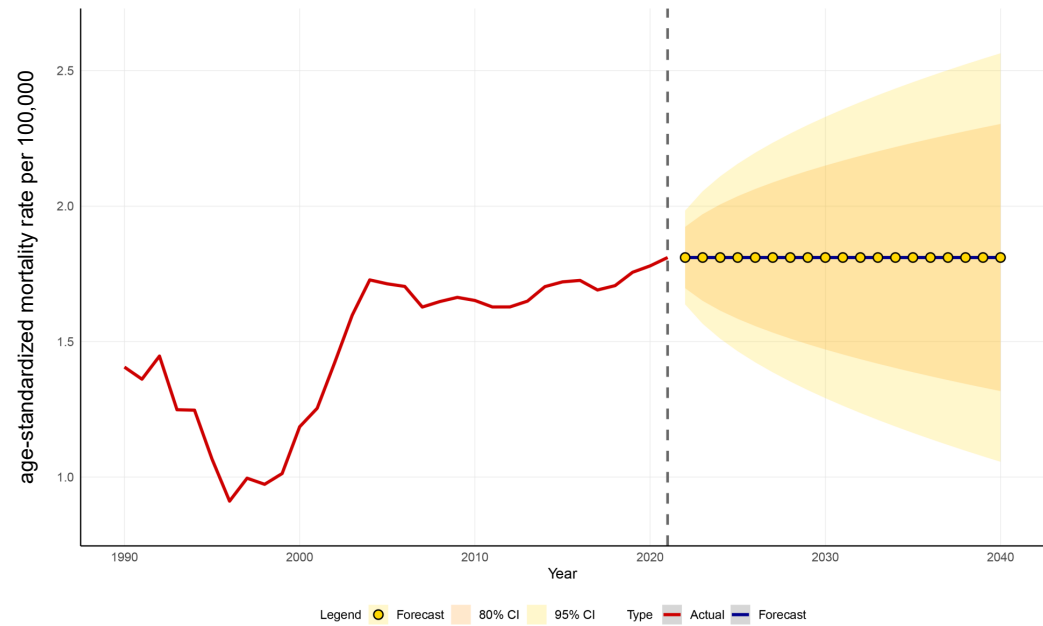**D**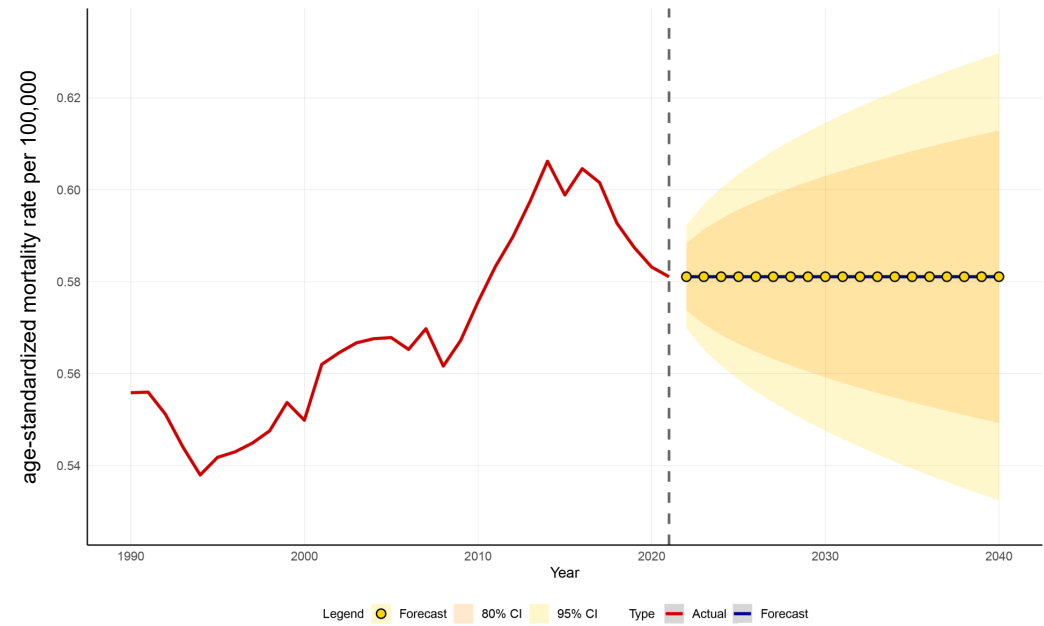

Supplement: Supplementary file 2 — Supplementary material 2 [file mmc2.pdf]

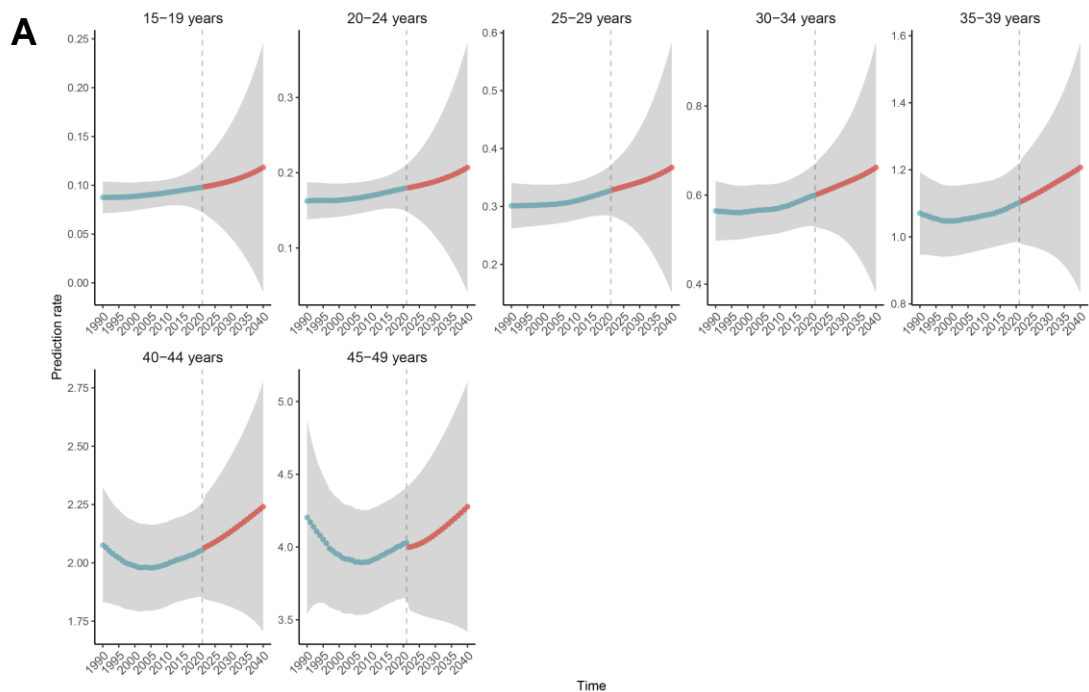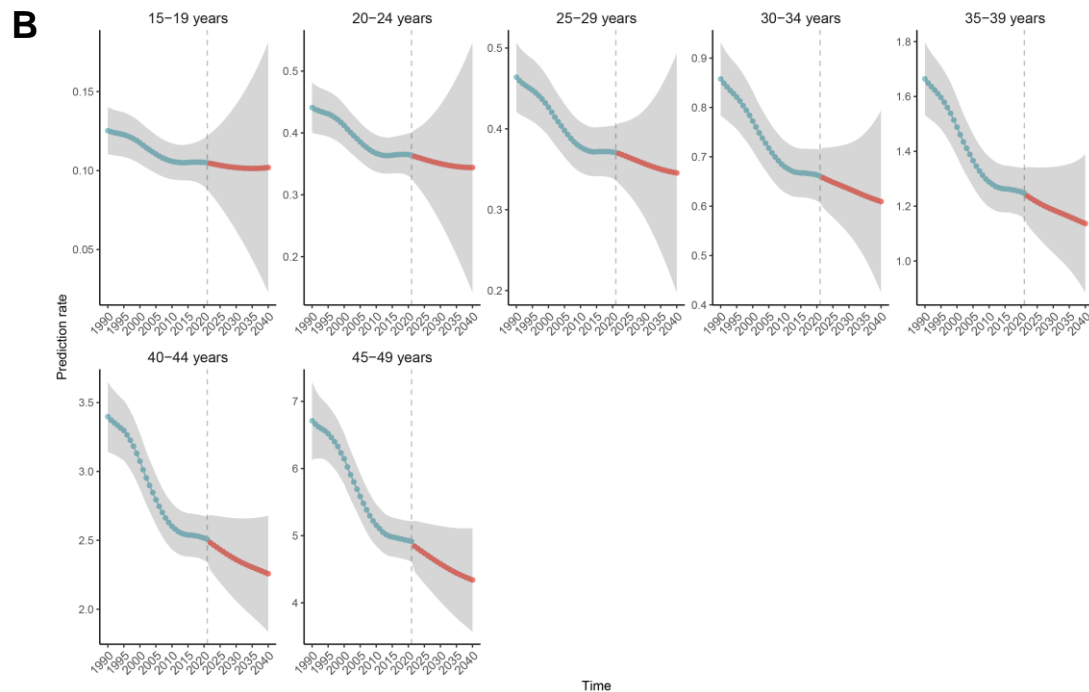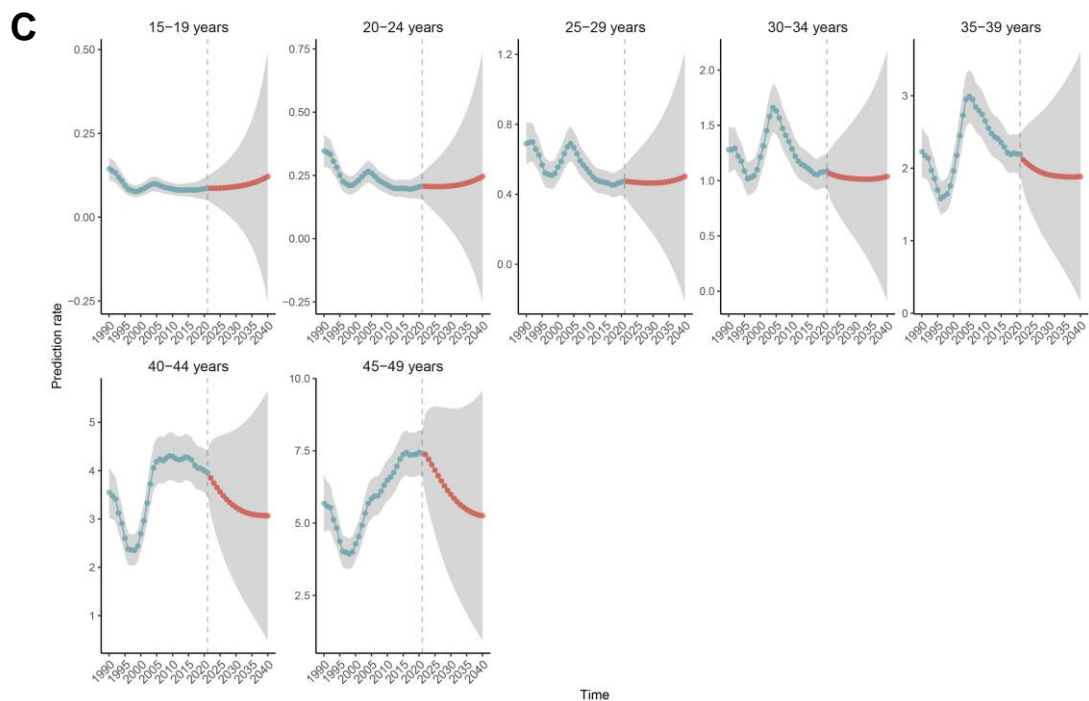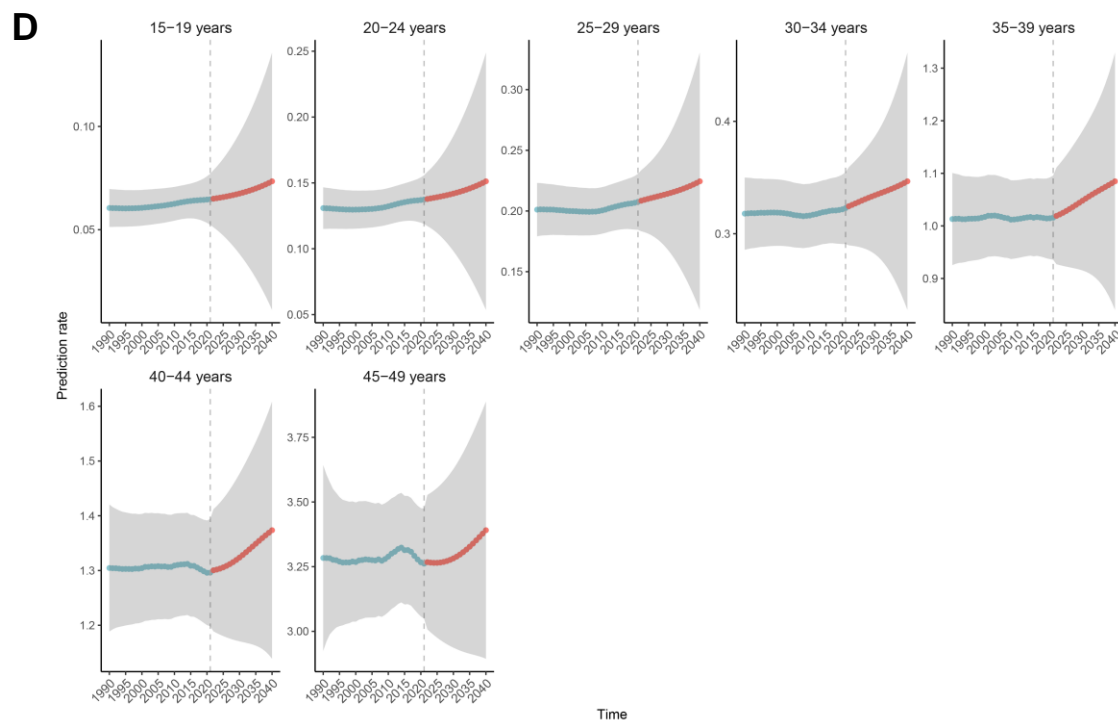

Supplement: Supplementary file 3 — Supplementary material 3 [file mmc3.pdf]
