# Supplementary material for: Trend analysis and epidemiological forecasting of colorectal Cancer mortality among reproductive-age women in sub-Saharan Africa
Source: Prev Med Rep. 2025 Jul 7;56:103167. doi: 10.1016/j.pmedr.2025.103167 (PMC12275479; doi:10.1016/j.pmedr.2025.103167)
Supplement: Supplementary file 4 — Supplementary material 4 [file mmc4.docx]

**Table S1** Deaths and age-standardized mortality rate of colorectal cancer among women of childbearing age in 1990 and 2021 and estimated annual percentage change from 1990 to 2021 in Sub-Saharan Africa countries

| Location | Death cases-1990  No. (95% UI) | 1990-ASMR-per 100000  (95% UI) | Death cases-2021  No. (95% UI) | 2021-ASMR-per 100000  (95% UI) | EAPC (95% CI) |
| --- | --- | --- | --- | --- | --- |
| Burkina Faso | 12.44(8.42,17.60) | 0.59(0.40,0.84) | 33.10(21.18,45.95) | 0.60(0.39,0.84) | 0.32(0.21,0.43) |
| Central African Republic | 7.30(3.90,11.62) | 1.14(0.61,1.81) | 16.08(8.52,29.44) | 1.16(0.62,2.13) | 0.04(-0.04,0.13) |
| Sao Tome and Principe | 0.12(0.08,0.15) | 0.45(0.31,0.60) | 0.34(0.22,0.51) | 0.61(0.40,0.91) | 0.81(0.51,1.12) |
| Democratic Republic of the Congo | 61.78(41.46,88.60) | 0.72(0.49,1.04) | 153.68(93.39,230.90) | 0.72(0.44,1.08) | 0.02(-0.16,0.20) |
| Ethiopia | 204.31(76.89,284.80) | 1.81(0.68,2.52) | 276.81(210.38,364.44) | 1.00(0.76,1.31) | -2.59(-2.91,-2.28) |
| Nigeria | 98.06(65.91,144.32) | 0.48(0.33,0.71) | 282.66(168.22,438.47) | 0.49(0.29,0.76) | 0.31(0.20,0.42) |
| Somalia | 25.13(12.09,43.83) | 1.48(0.71,2.59) | 57.51(27.41,95.11) | 1.19(0.57,1.97) | -1.21(-1.41,-1.02) |
| Gabonese Republic | 2.62(1.40,4.18) | 1.18(0.63,1.89) | 6.43(3.97,9.76) | 1.31(0.81,1.99) | 0.32(0.03,0.62) |
| Mauritania | 2.90(1.92,4.17) | 0.62(0.41,0.89) | 7.69(5.09,11.39) | 0.72(0.47,1.06) | 0.52(0.39,0.65) |
| Eswatini | 2.16(1.56,2.93) | 1.11(0.80,1.50) | 5.31(2.75,8.62) | 1.68(0.87,2.74) | 1.69(0.95,2.43) |
| Lesotho | 2.54(1.63,3.88) | 0.67(0.43,1.03) | 8.07(4.49,13.91) | 1.59(0.89,2.74) | 4.17(3.37,4.97) |
| Angola | 20.51(13.48,28.96) | 0.89(0.59,1.26) | 69.15(42.01,110.26) | 0.90(0.55,1.44) | 0.19(0.02,0.36) |
| Benin | 5.15(3.68,7.04) | 0.47(0.34,0.64) | 17.15(10.71,26.69) | 0.53(0.33,0.82) | 0.48(0.36,0.59) |
| Botswana | 3.04(1.86,4.74) | 0.95(0.58,1.47) | 7.57(4.35,14.23) | 1.11(0.64,2.09) | 1.29(0.59,2.00) |
| Burundi | 15.56(9.27,23.09) | 1.24(0.74,1.83) | 26.45(17.85,38.83) | 0.85(0.57,1.24) | -1.90(-2.16,-1.65) |
| Cabo Verde | 0.34(0.25,0.47) | 0.43(0.31,0.59) | 1.06(0.72,1.51) | 0.70(0.48,1.00) | 1.11(0.52,1.71) |
| Cameroon | 17.35(12.45,23.73) | 0.73(0.52,1.00) | 60.02(37.56,90.25) | 0.77(0.48,1.15) | 0.34(0.26,0.41) |
| Chad | 6.64(4.62,9.67) | 0.50(0.35,0.73) | 24.51(15.34,35.57) | 0.63(0.40,0.92) | 1.06(0.93,1.20) |
| Côte d'Ivoire | 11.73(8.07,16.01) | 0.43(0.29,0.58) | 33.64(21.17,49.30) | 0.51(0.32,0.74) | 1.04(0.87,1.21) |
| Djibouti | 1.00(0.59,1.60) | 1.02(0.60,1.63) | 4.39(2.41,7.69) | 1.37(0.75,2.39) | 0.91(0.77,1.05) |
| Equatorial Guinea | 0.99(0.60,1.51) | 1.00(0.60,1.53) | 3.73(1.94,6.68) | 1.02(0.53,1.83) | 0.15(-0.02,0.32) |
| Ghana | 30.78(19.59,44.24) | 0.87(0.56,1.25) | 82.03(54.55,121.94) | 0.90(0.60,1.34) | 0.01(-0.09,0.11) |
| Guinea | 7.00(4.86,9.23) | 0.51(0.36,0.68) | 15.89(10.08,26.60) | 0.48(0.30,0.80) | -0.21(-0.26,-0.15) |
| Guinea-Bissau | 2.22(1.28,3.26) | 0.95(0.55,1.40) | 5.76(3.60,8.75) | 1.10(0.69,1.67) | 0.57(0.47,0.66) |
| Kenya | 36.55(26.42,45.67) | 0.71(0.51,0.88) | 129.38(94.60,183.13) | 0.97(0.71,1.38) | 1.38(1.24,1.52) |
| Liberia | 2.66(1.84,3.92) | 0.48(0.33,0.71) | 10.28(5.62,16.02) | 0.74(0.40,1.15) | 1.17(0.84,1.50) |
| Madagascar | 34.91(24.27,48.17) | 1.30(0.90,1.79) | 85.85(58.16,120.13) | 1.19(0.80,1.66) | -0.26(-0.39,-0.12) |
| Malawi | 13.04(9.64,17.19) | 0.58(0.43,0.76) | 25.33(15.33,42.72) | 0.51(0.31,0.85) | -0.61(-0.73,-0.49) |
| Mali | 16.92(12.56,22.82) | 0.88(0.65,1.19) | 37.01(24.21,61.30) | 0.68(0.44,1.13) | -0.88(-0.95,-0.81) |
| Mozambique | 6.13(4.23,8.52) | 0.19(0.13,0.27) | 14.28(8.67,23.04) | 0.19(0.11,0.30) | 0.33(0.10,0.56) |
| Namibia | 2.49(1.84,3.28) | 0.73(0.54,0.97) | 5.18(3.21,8.07) | 0.78(0.49,1.22) | -0.10(-0.42,0.22) |
| Rwanda | 26.90(16.76,36.91) | 1.66(1.03,2.27) | 35.71(19.96,57.07) | 1.01(0.57,1.62) | -2.60(-3.05,-2.14) |
| Senegal | 9.94(7.02,13.53) | 0.58(0.41,0.79) | 27.91(18.72,41.01) | 0.72(0.48,1.05) | 0.90(0.71,1.08) |
| Sierra Leone | 3.78(2.38,5.70) | 0.38(0.24,0.57) | 13.62(8.55,20.20) | 0.60(0.38,0.89) | 1.88(1.69,2.07) |
| South Africa | 153.52(133.71,175.95) | 1.59(1.39,1.82) | 274.84(225.35,336.35) | 1.77(1.46,2.17) | 1.07(0.50,1.65) |
| South Sudan | 12.88(7.72,19.96) | 0.99(0.60,1.54) | 30.70(18.93,46.17) | 1.32(0.82,1.99) | 1.02(0.52,1.53) |
| Republic of the Congo | 6.39(3.65,9.43) | 1.13(0.65,1.67) | 21.90(13.70,32.67) | 1.53(0.96,2.28) | 0.94(0.75,1.14) |
| Gambia | 0.65(0.43,0.90) | 0.28(0.19,0.40) | 2.27(1.49,3.41) | 0.37(0.24,0.55) | 0.60(0.32,0.88) |
| Niger | 8.98(5.80,13.57) | 0.52(0.33,0.78) | 25.31(15.07,38.44) | 0.47(0.28,0.72) | -0.15(-0.24,-0.06) |
| Uganda | 33.03(21.32,47.10) | 0.86(0.55,1.22) | 110.66(68.77,164.73) | 1.06(0.66,1.58) | -0.24(-0.67,0.19) |
| Zambia | 25.80(18.26,34.50) | 1.40(0.99,1.88) | 71.95(36.98,159.60) | 1.46(0.75,3.24) | -0.01(-0.29,0.28) |
| Zimbabwe | 23.11(17.66,30.90) | 0.96(0.73,1.29) | 92.07(58.35,136.29) | 2.27(1.44,3.36) | 4.13(3.14,5.13) |
| Eritrea | 11.49(7.12,16.48) | 1.46(0.90,2.09) | 25.36(16.17,38.57) | 1.53(0.98,2.33) | 0.38(0.26,0.51) |
| Togolese Republic | 4.72(3.36,6.51) | 0.55(0.39,0.76) | 16.41(9.57,26.14) | 0.76(0.44,1.21) | 1.35(1.15,1.54) |
| Comoros | 1.45(0.67,2.08) | 1.38(0.64,1.97) | 2.74(1.53,4.34) | 1.40(0.79,2.22) | -0.29(-0.66,0.09) |
| Tanzania | 62.22(46.12,80.59) | 1.04(0.77,1.34) | 149.98(97.11,225.66) | 1.01(0.65,1.52) | -0.04(-0.18,0.09) |

**Abbreviations:** ASMR, age-standardized mortality rate; EAPC, estimated annual percentage change; UI, Uncertainty Interval; CI, Confidence Interval.
