# Supplementary material for: Trend analysis and epidemiological forecasting of colorectal Cancer mortality among reproductive-age women in sub-Saharan Africa
Source: Prev Med Rep. 2025 Jul 7;56:103167. doi: 10.1016/j.pmedr.2025.103167 (PMC12275479; doi:10.1016/j.pmedr.2025.103167)
Supplement: Supplementary file 5 — Supplementary material 5 [file mmc5.docx]

**Table S2** Decomposition analysis of the changes in mortality rates for colorectal cancer among women of childbearing age in Sub-Saharan Africa countries

| Location | Aging (Percentage) | Population (Percentage) | Epidemiological change (Percentage) |
| --- | --- | --- | --- |
| Burkina Faso | -0.17（-0.8％) | 19.28（93.31％) | 1.55（7.49％) |
| Central African Republic | -1.32（-15.02％) | 10.31（117.48％) | -0.22（-2.46％) |
| Sao Tome and Principe | -0.03（-14.89％) | 0.23（104.42％) | 0.02（10.47％) |
| Democratic Republic of the Congo | 3.04（3.31％) | 92.56（100.72％) | -3.7（-4.03％) |
| Ethiopia | -3.64（-5.02％) | 236.33（325.99％) | -160.2（-220.97％) |
| Nigeria | 39.23（21.25％) | 159.03（86.14％) | -13.65（-7.4％) |
| Somalia | 3.33（10.28％) | 36.2（111.77％) | -7.14（-22.05％) |
| Gabonese | 0.19（4.99％) | 3.9（102.5％) | -0.29（-7.49％) |
| Mauritania | -0.19（-4％) | 4.49（93.83％) | 0.49（10.18％) |
| Eswatini | -1.2（-38.2％) | 3.26（103.73％) | 1.08（34.47％) |
| Lesotho | -4.23（-76.48％) | 4.83（87.35％) | 4.93（89.13％) |
| Angola | 13.47（27.69％) | 36.23（74.47％) | -1.05（-2.16％) |
| Benin | 2.12（17.69％) | 9（75.03％) | 0.87（7.28％) |
| Botswana | 0.69（15.14％) | 4.58（101.19％) | -0.74（-16.33％) |
| Burundi | -0.59（-5.43％) | 19.6（179.82％) | -8.11（-74.39％) |
| Cabo Verde | 0.04（5％) | 0.57（79.85％) | 0.11（15.16％) |
| Cameroon | 10.99（25.76％) | 31.03（72.72％) | 0.65（1.53％) |
| Chad | 1.06（5.95％) | 12.3（68.87％) | 4.5（25.18％) |
| Côte d'Ivoire | 2.18（9.94％) | 18.92（86.33％) | 0.82（3.73％) |
| Djibouti | 1.31（38.77％) | 2.07（61.26％) | 0（-0.03％) |
| Equatorial Guinea | 0.79（28.75％) | 1.86（67.73％) | 0.1（3.52％) |
| Ghana | 8.67（16.91％) | 47.86（93.36％) | -5.26（-10.27％) |
| Guinea | -0.32（-9.01％) | 3.41（96.29％) | 0.45（12.72％) |
| Guinea-Bissau | -1.22（-13.75％) | 10.02（112.72％) | 0.09（1.02％) |
| Kenya | 13.6（14.66％) | 65.91（71％) | 13.32（14.34％) |
| Liberia | 1.05（13.72％) | 5.05（66.28％) | 1.52（20％) |
| Madagascar | 9.43（18.51％) | 52.24（102.55％) | -10.73（-21.06％) |
| Malawi | -2.39（-19.41％) | 17.36（141.22％) | -2.68（-21.81％) |
| Mali | 0.81（4.04％) | 23.83（118.63％) | -4.56（-22.67％) |
| Mozambique | -1.34（-16.39％) | 8.9（109.12％) | 0.59（7.27％) |
| Namibia | -0.22（-8.12％) | 3.42（127.06％) | -0.51（-18.93％) |
| Rwanda | -0.26（-2.93％) | 30.88（350.57％) | -21.82（-247.63％) |
| Senegal | 0（-0.01％) | 15.84（88.14％) | 2.13（11.87％) |
| Sierra Leone | -0.69（-6.96％) | 6.97（70.86％) | 3.55（36.1％) |
| South Africa | -44.39（-36.59％) | 195.86（161.45％) | -30.15（-24.86％) |
| South Sudan | -3.19（-17.9％) | 18.96（106.43％) | 2.04（11.47％) |
| Republic of the Congo | 3.36（21.7％) | 11.37（73.34％) | 0.77（4.96％) |
| Gambia | 0.24（15.02％) | 1.16（71.56％) | 0.22（13.42％) |
| Niger | 2.24（13.74％) | 14.35（87.87％) | -0.26（-1.6％) |
| Uganda | 8.81（11.35％) | 57.88（74.57％) | 10.93（14.08％) |
| Zambia | 6.81（14.76％) | 41.06（88.98％) | -1.72（-3.74％) |
| Zimbabwe | -12.37（-17.94％) | 46.16（66.93％) | 35.17（51.01％) |
| Eritrea | -1.75（-12.59％) | 16.25（117.09％) | -0.62（-4.5％) |
| Togolese Republic | 1.87（16％) | 8.43（72.07％) | 1.39（11.92％) |
| Comoros | -0.24（-18.93％) | 1.91（148.33％) | -0.38（-29.39％) |
| Tanzania | 11.02（12.56％) | 92.03（104.87％) | -15.3（-17.43％) |
